# In Vitro Methods for Evaluating Drug Release of Vaginal Ring Formulations—A Critical Review

**DOI:** 10.3390/pharmaceutics11100538

**Published:** 2019-10-16

**Authors:** Katharina Tietz, Sandra Klein

**Affiliations:** Center of Drug Absorption and Transport, Institute of Biopharmaceutics and Pharmaceutical Technology, Department of Pharmacy, Felix-Hausdorff-Str. 3, University of Greifswald, 17489 Greifswald, Germany; Katharina.Tietz@uni-greifswald.de

**Keywords:** intravaginal ring, dissolution method, drug release

## Abstract

The vagina is a promising site for both local and systemic drug delivery and represents an interesting administration route for compounds with poor oral bioavailability. Whereas most of the currently marketed dosage forms were designed as immediate release formulations, intravaginal rings (IVRs) offer the possibility of a controlled vaginal drug delivery over several weeks or months. For a long time, the development of IVRs was limited to steroid-releasing formulations. Recently, IVRs have witnessed a surge of new interest as promising delivery systems for microbicides. Therefore, various novel IVR designs have been introduced. To ensure that only safe and effective IVRs will be administered to patients, it is important to properly distinguish between IVRs with desired and undesired release performance. In vitro methods for evaluating drug release of IVRs that present with sufficient predictive capacity for in vivo drug release, and discriminatory power with regard to IVRs quality, are an essential tool for this purpose. The objective of the present review article is to present the current status of in vitro drug release testing of IVRs and to critically discuss current compendial and non-official in vitro drug release methods with regard to their discriminatory power and in vivo predictivity.

## 1. Introduction

The female vagina is well known as a genital organ with functions related to conception. Whereas for many years, it was not considered as a site for systemic drug administration and dosage forms administered to the vagina were intended for local action, more recently, it became obvious that the vaginal route also offers the potential of delivering drugs for systemic action and uterine targeting. Thus, nowadays, the vagina is also a promising site of systemic drug delivery and represents an interesting administration route for compounds with poor oral bioavailability, e.g., proteins [1]. Currently, different types of vaginal dosage forms for both local and systemic action are available on the market or in clinical development. Commonly used vaginal dosage forms include classical liquid, semisolid and solid formulations such as solutions, emulsions and suspensions, ointments, creams and gels, suppositories, pessaries, inserts, tablets and capsules. In the more recent past some novel types of formulations including foams and films, and more specialized administration devices such as tampons and vaginal rings were developed. Whereas most of the currently marketed dosage forms were designed as immediate release (IR) formulations, vaginal rings, also called intravaginal rings (IVRs) offer the possibility of a controlled vaginal drug delivery over several weeks or months. The first IVRs were developed as alternatives for long-acting parenterals to provide continuous medication during a predetermined medication period via the vagina in female mammals. The first patent application for an IVR was filed in the late 1960s [2], but it took until the early 1990s before the first IVRs reached the market. The very first marketed formulation was a contraceptive IVR containing progesterone (Progering^®^). Shortly thereafter, IVRs for hormone replacement became available (Femring^®^, Estring^®^). In 2002, the first combined contraceptive IVR, i.e., the NuvaRing^®^ which releases 120 µg etonogestrel and 15 µg of ethinyl estradiol per day over a 3-week period of use, was approved by the United States Food and Drug Administration (FDA). Currently, several long-acting steroid-releasing IVRs that are used in hormone replacement therapy or birth control are available on the market as seen in Table 1.

Whereas for a long time IVR development was limited to steroid-releasing IVRs, in recent years IVRs have witnessed a surge of new interest as promising delivery systems for microbicides, which represent compounds or formulations that can prevent the transmission of the human immunodeficiency virus (HIV) and other sexually transmitted infections (STIs) [7,8]. Currently, there is also a big interest in the development of multipurpose prevention devices, i.e., IVRs containing microbicides and contraceptives to provide protection from both STIs and unintended pregnancy [9,10,11,12]. Sub-Saharan Africa remains the region most affected by HIV and women are at a greater physiological risk of contracting HIV than men. Overall, HIV/AIDS is the leading cause of death globally in women aged 15–44 [13]. Since every day thousands of women are newly infected with HIV, there is a huge need for novel treatment and formulation strategies that prevent transmission during sexual intercourse. Vaginal microbicides promise to address a central gap in current HIV-prevention strategies. Therefore, the efforts in developing novel microbicide formulations are supported by many non-profit organizations such as the World Health Organization (WHO), the Population Council or the Bill and Melinda Gates Foundation. There are no approved microbicides available yet, but currently, it looks like the first microbicide to be submitted for regulatory approval will be a silicone-based membrane-type IVR developed by the International Partnership for Microbicides (IPM). This IVR releases dapivirine for local action in the vagina over the duration of one month [14].

**Figure 1:**
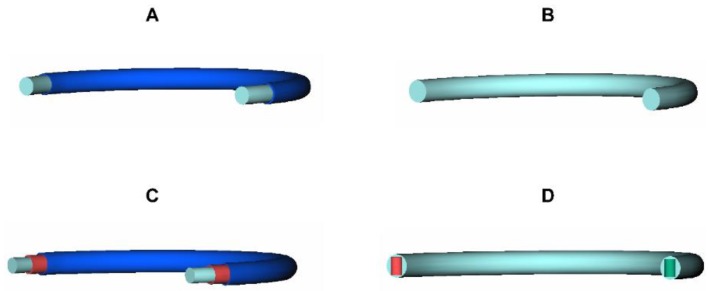
Different IVR designs: (**A**) Reservoir-type with drug-loaded core and drug-free membrane; (**B**) Matrix-type with drug dispersed in the polymer matrix; (**C**) Sandwich-type with drug-free core, drug loaded layer and drug-free membrane; and (**D**) Pod-type IVR with drug-free ring backbone with different pods inserted.

Based on the intended use, the duration of action and the number of drugs to be administered, various IVR designs are available. An overview of the most frequently used designs is given in Figure 1. Additional IVR designs can be found in the literature, e.g., in [15]. Figure 1A shows a schematic view of a reservoir-type IVR, as it is used in the NuvaRing^®^, the Femring^®^ or the Estring^®^ device. This IVR type is composed of a drug-loaded core covered by a drug-free membrane [16]. Drug release of reservoir-type IVRs is usually controlled by the nature and thickness of the polymer membrane. Figure 1B shows a schematic view of a matrix-type IVR, which is the simplest IVR design and used for the Progering^®^ and the Fertiring^®^. In the matrix-type IVR, the drug is homogeneously dispersed/dissolved in the matrix polymer and drug release rates are proportional to both the drug loading and the surface area of the device [16]. A schematic view of a sandwich-type IVR is depicted in Figure 1C. A sandwich type IVR consists of a drug-free core surrounded by a drug-loaded polymer layer, which is covered by a drug-free polymer layer. As in the reservoir-type ring, the outer layer is intended to control drug release and can either be made of the core polymer or another polymer that provides the desired release rate [16]. An innovative ring design, which is called the pod-type IVR, is shown in Figure 1D. Pod-type IVRs represent a modular design which consists of a drug-free scaffold with individual polymer-coated drug cores (pellets or tablets), referred to as pods, that are positioned into premolded, evenly spaced cavities [16,17]. Multiple pods containing different types of drugs can be placed into a single IVR. Drug release from the IVRs is determined by the individual coatings of the pods and by the characteristics (e.g., number, geometry, and cross-sectional area) of the delivery channels in the impermeable IVR structure. Consequently, the pod-type IVR design allows the simultaneous release of two or more drugs with release rates that can be titrated for each individual drug or pod [17].

Since in 1970 the patent “Medicated devices and methods” was granted, the shape of IVRs did not change [2]. At this time, silicone elastomers were very popular and were used for manufacturing the first IVRs containing steroids. An advantage of silicone elastomers is their excellent biocompatibility, but a major disadvantage is the limited availability of medical grade materials. Therefore, within the following decades other polymers, especially thermoplastic polymers, became more important in manufacturing IVRs. Frequently used thermoplastic polymers for manufacturing IVRs include polyethylene vinyl acetate (PEVA) and polyurethane (PU). IVRs made of these polymers are usually prepared by injection molding or hot-melt extrusion [18,19,20]. In the recent past, biodegradable polymers, such as polycaprolactone (PCL) were evaluated as microporous matrices for controlled vaginal delivery of antiviral microbicides, and are likely to be further investigated in future studies [21,22,23].

All of the currently marketed IVRs shown in Table 1 rely on a permeation-controlled drug release mechanism [24], which can be distinguished into three discrete and consecutive steps: (i) drug solvation in the surrounding polymer, (ii) molecular diffusion of the solvated drug molecules within the polymer, and (iii) partition of the drug from the ring surface into vaginal fluid [15]. Consequently, the driving force for drug release from IVRs is passive diffusion down concentration gradients that exist from within the device to the fluid in the vaginal cavity [15]. However, although diffusion is the driving force for drug release of all currently marketed IVR formulations, drug release profiles obtained from membrane-type and matrix-type IVRs are different in nature, and even for the same drug vary with several formulation parameters like drug load, membrane thickness and polymer type(s). With the pod-IVRs and some other novel formulation approaches, in the recent past other mechanisms of drug release beyond permeation control have been investigated, and some of these formulations are likely to reach the market in the near future [15]. To ensure that only safe and effective IVRs will be administered to patients, it is important to properly distinguish between IVRs to ensure the desired in vivo drug release. For IVRs containing multiple drugs, this applies to each individual compound. In vitro methods for evaluating drug release of IVRs that present with sufficient predictive capacity for in vivo drug release, and discriminatory power with regard to IVRs with different release rates, are thus an essential tool in different stages of formulation development and in quality control (QC) of finished products.

The objective of the present review article is to present the current status of in vitro drug release testing of IVRs and to critically discuss current compendial and non-official in vitro drug release methods with regard to their discriminatory power and in vivo predictivity.

## 2. Current In Vitro Methods for Evaluating Drug Release of Intravaginal Rings

### 2.1. Compendial Methods

IVRs are not described in international pharmacopoeia, such as the United States (USP) [25], the European (Ph.Eur.) [26], the Japanese (JP) [27]. or the International Pharmacopoeia (IP) [28]. According to the USP, IVRs could be regarded as vaginal systems. According to the USP General Chapter <1151> systems are preparations of drug substance(s) in carrier devices, which are applied topically or inserted into body cavities. The drug substances contained in these systems are designed to be released in a controlled manner over a specified period of time or the drug substance is released based on its concentration in the formulation. After use, the carrier device is removed [25]. In the scientific literature, IVRs are described as torus-shaped, flexible, and elastomeric drug delivery devices that can provide long-term controlled drug release for local or systemic action [3,29,30,31].

Since in none of the major international pharmacopoeia, a monograph for IVRs is available and IVRs are also not mentioned in the general pharmacopoeial chapters on dissolution/drug release testing, it is obvious that compendial dissolution methods do not exist.

### 2.2. Methods Described in Regulatory Guidances

Neither in the United States, nor in Europe or Japan, is a regulatory guidance on drug release testing of IVRs available. This is somewhat surprising, since as a result of the NuvaRing^®^ patent expiry particularly in Europe, in the recent past, numerous generic IVRs have been developed and were released to the market. Both an increasing number of marketed products, and the numerous IVR formulations that are currently in development, indicate the need for appropriate in vitro drug release methods for both in vivo performance prediction and QC.

The Dissolution Methods Database in which the FDA provides information on dissolution methods, presently recommended by the Division of Biopharmaceutics, Office of Pharmaceutical Quality to aid industry personnel in developing generic drug products, lists a dissolution method for an estradiol-containing IVR. Moreover, the need for developing an appropriate in vitro drug release method for a combined contraceptive ethinyl estradiol/etonogestrel IVR is indicated as shown in Table 2 [32].

The method recommended for drug release testing of estradiol IVRs is a quite simple method and does not even require an official dissolution/drug release apparatus. The dissolution medium to be applied is isotonic saline solution, which is a medium that does not address fluid properties (pH, buffer capacity, surface tension etc.) at the administration site. It should be clear that this test method was mainly designed for QC, i.e., to ensure that the IVR is able to release estradiol with a specific release pattern and within a certain time frame and to discriminate between good and bad batches rather than for simulating in vivo conditions. In some cases, it is possible to correlate in vitro drug release obtained with such simple in vitro methods with in vivo drug release profiles, but this is then usually done on a retrospective basis. By contrast, for a proper prediction of the in vivo performance of an IVR, the physiological conditions that are relevant to in vivo drug release should be properly addressed.

The NuvaRing^®^, a combined contraceptive ethinyl estradiol/etonogestrel IVR was approved by FDA in 2002. In the approval documents test of this IVR, conditions for drug release testing were provided by the manufacturer. Unfortunately, the detailed dissolution method is not accessible by the public. However, the manufacturer indicates that the test conditions relate to US patent US7357046B2 “Method for dissolution testing of a pharmaceutical delivery device”, which relates to a particular method for dissolution testing of an annular pharmaceutical delivery device [33]. The approval documents of NuvaRing^®^ indicate that these test conditions were regarded as appropriate for obtaining an in vitro-in vivo correlation (IVIVC) for this particular formulation. Even though in the cited patent there is also no detailed information provided, it is clear that the applied in vitro method was a quite simple setup with an inert vessel, a device holder that prevents floating of the IVR, a stirrer to agitate the dissolution medium, and a standard dissolution medium. The NuvaRing^®^ in vitro drug release method was assessed by FDA quite some time ago and recently several generic formulations of this IVR have been introduced, however to date, a dissolution method recommendation for formulation screening of combined contraceptive ethinyl estradiol/etonogestrel IVRs was not published.

### 2.3. Methods Described in the Relevant Literature

We conducted a detailed literature review of publications about IVRs with a special focus on research performed in the field of in vitro drug release testing of IVRs. It became obvious, that, independent of the type of the IVR, in most cases, very simple in vitro setups are applied. Typically, the IVR is added to a flask/vessel and immersed in dissolution medium. The medium is agitated by shaking or stirring and the media temperature is controlled throughout the entire experiment. Samples are removed at predetermined time points and either the sampling volume or the entire media volume is replaced by fresh medium. Whereas the same general test setup was used in many of the assessed literature reports, other test conditions, i.e., media composition, volume and temperature, agitation speed, and sampling frequency were quite different among the different studies. Table 3 gives an overview of a selection of in vitro test methods with essentially different designs. In the following sections, the methodological approaches and dissolution media described in the literature will be discussed in more detail.

### 2.4. Methodological Approaches

**Figure 2:**
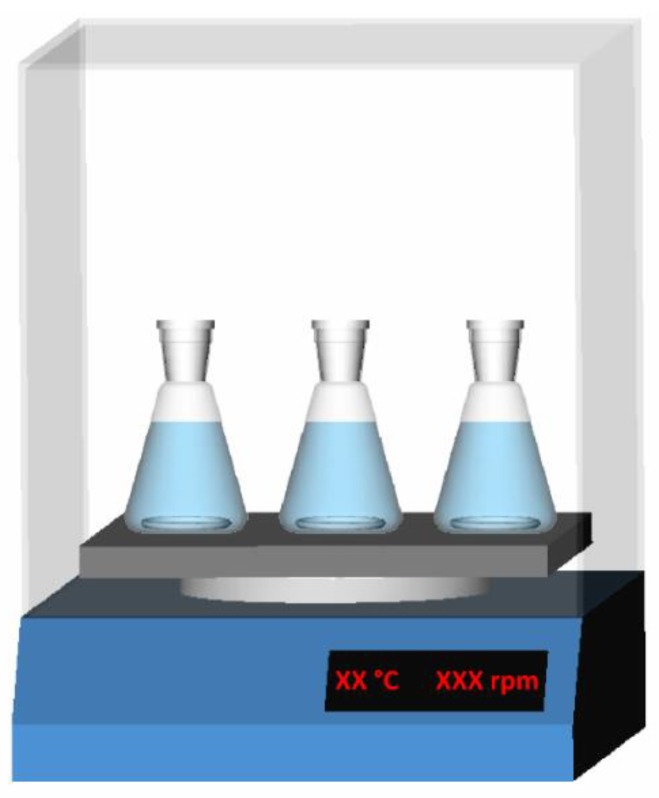
Schematic view of a shake-flask setup used for determining drug release of IVRs.

The so-called shake-flask method is often used to determine drug release from IVRs. A common setup for a shake-flask experiment is given in Figure 2.

Shake-flask experiments are usually performed in an incubator shaker setup. Experiments are performed with IVRs for human or preclinical use, or ring segments. An individual IVR or a ring segment is added to a specific volume of a (pre-heated) dissolution medium in a flask (entire ring), polypropylene tube [21], scintillation vial [34], glass bottle [35], or a sealed flask [36], and placed into an incubator shaker. The applied media volumes differ between experiments performed with ring segments, rings for preclinical use, and human IVRs. When testing ring segments, media volumes are usually small (5–20 mL per segment). The highest media volume reported for testing entire IVRs is as high as 1000 mL [63,64,65]. Preclinical in vivo drug release studies are often performed in macaques. Since IVRs for human use do not fit into the vaginal cavity of female macaques, IVRs with a smaller diameter are used for these experiments. Consequently, the volumes of test media used for in vitro drug release experiments with macaque-sized IVRs are often significantly smaller than those used in human IVR experiments [24,37,38,39,40]. The reported agitation speeds in shake-flask experiments range from 60 rpm [31,35,36,37,38,39,40,41,42,43,44,45,46,47,48,70,75,78,79,80] through 80 rpm [51,52,53,54,73], 100 rpm [10,56,57,58,71] and 110 rpm [17] to 130 rpm [34,49,50,81,82].

Besides the incubator shake-flask method, several other in vitro setups have been described. Gupta et al. used a water bath shaker agitated at 64 ± 2 rpm for determining dapavirine release from a polyurethane IVR [59]. Van Laarhoven et al. used an “Automated release control system” in which the medium was stirred with an agitation rate of 750 rpm for studying etonogestrel and ethinyl estradiol release from combined contraceptive IVR formulations [60,61,62]. Some research groups also used compendial apparatuses. Helbling et al. used the USP apparatus 1 (basket) with the aim of determining progesterone release from the commercial Progering^®^ and an EVA-based IVR containing progesterone, and to further identify the main factors that influence the drug release rate of EVA-made IVRs [63,64,65]. In their experiments, the rings were placed in stainless steel baskets agitated at 25 rpm or 100 rpm [63]. 1000 mL of an ethanol:water mixture (20% ethanol *v*/*v*) was used as the dissolution medium. At pre-determined time points, samples of 5 mL were taken and the sampling volume was replaced by fresh medium [64]. In a subsequent study, Helbling et al. used the same experimental setup to evaluate different cellulose membranes for their potential to eliminate the extensive burst release of progesterone (20–40% progesterone release within a short initial burst phase) that had been observed in the previous studies with EVA-based rings [65].

Externbrink et al. used a small volume USP apparatus 7 (400-DS, Agilent Technologies) to study drug release of the NuvaRing^®^ with the aim of establishing accelerated test conditions that can provide real-time release profiles within a very short test duration [49]. Experiments were performed with NuvaRing^®^ segments that, prior to the experiments at both ends, had been sealed with Loctite^®^ acrylate glue to ensure that drug release was solely controlled by the ring membrane. Release experiments were performed at different temperatures, i.e., 37, 45, 50 and 55 °C. 10 mL of Vaginal Fluid Simulant (VFS) with 0.1% sodium azide was used as release medium. Sampling was performed automatically and sampling intervals were adjusted at elevated temperatures. By weighing entire rings and all corresponding ring segments, drug release profiles obtained for the individual segments were standardized to that of the entire ring via the mass ratio (segment/entire ring). Using the instrumental setup, but acetate buffer pH 4.50 as release medium, Externbrink et al. also evaluated etonogestrel and ethinyl estradiol release of a matrix-type IVR in real-time and accelerated test conditions [34]. In both studies the shake-flask method was used as reference method. Eder et al. studied etonogestrel and ethinyl estradiol release from cylindrical reservoir co-extrudates made of different types of PEVA [50]. They placed the individual co-extrudate prototypes in an USP I basket to avoid floating during drug release studies. The baskets were then transferred into flasks filled with 30 mL of pre-heated MilliQ water, which were placed in an incubator shaker at 37.0 °C and agitated at 130 rpm. Samples were collected in 24 h intervals and a full media change was performed after each sampling.

Recently, McBride et al. presented a novel ring design, the so called exposed-core IVR comprising one or more drug-loaded hydroxypropyl methylcellulose (HPMC) cores exposed to the external environment via orifices or windows in an overmolded silicone sheath [48]. Drug release of these novel IVR types was studied in a very simple setup, i.e., poylpropylene tubes containing 15 mL of MilliQ water of 37 °C. The tubes were placed in an incubator shaker and agitated at 60 rpm throughout the experiment. Samples were taken periodically with complete media replacement.

In the course of developing PEVA-based disulfiram-loaded vaginal rings for the localized treatment of cervical cancer, Boyd et al. evaluated a novel method that had been designed to better mimic conditions in the vagina, i.e., the aqueous environment in the vaginal vault which is surrounded by hydrophobic tissue [46]. A sealed latex balloon simulated the vaginal wall and was filled with 20 mL water to mimic vaginal fluid. First, the IVR was immersed in the aqueous phase inside the balloon (inner compartment). Then, then the balloon was sealed and placed into a flask containing 100 mL of 2% sodium dodecyl sulfate (SDS) solution (outer compartment). Subsequently, the flask was placed into an orbital shaking incubator and agitated at 37 °C and 60 rpm. The experiment was run for 14 days and samples were taken daily from both the inner and outer compartment. At each sampling point, a full media change was performed for both compartments.

### 2.5. Release Media

In vitro drug release of IVRs is either performed for QC or for predicting in vivo drug release. Dissolution methods to be applied in QC should be discriminating and robust and need to ensure that drug release is controlled by the dosage form rather than the test conditions. A biopredictivity is not required. Therefore, QC methods are usually of simple design comprising a standard test apparatus and simple aqueous dissolution media. As stated before, only one “official” QC method for an IVR, describing the use of 0.9% saline solution as a dissolution medium for an estradiol IVR, can be found in the FDA dissolution methods database. For all other marketed IVRs, there is no information on officially accepted dissolution media available. However, other media used for evaluating drug release of IVRs can be found in the scientific literature. The least complex medium used for QC purposes is water. Due to its lacking buffer capacity in pharmaceutical dissolution, testing water is also one of the least reliable media for dissolution testing, and its use in in vitro drug release testing of IVRs is not very common [46,48,50,61,66,67]. Sink conditions are an essential prerequisite for in vitro dissolution test methods used in QC. As the fluid volume applied in in vitro drug release testing of IVRs is usually small, but many of the relevant APIs are poorly soluble, selection of an appropriate dissolution medium is essential for ensuring sink conditions throughout the experiment. The addition of surfactants to aqueous dissolution media can be an appropriate means for this purpose. Boyd et al. acquired in vitro drug release data of their disulfiram-loaded PEVA vaginal rings in a 2% SDS solution [46]. Externbrink et al. added 0.3% SDS to their release medium to increase the solubility of etonogestrel and ethinyl estradiol [34]. A rarely used surfactant in dissolution testing is benzalkonium chloride (BAC). Malcolm et al. studied in vitro drug release from steroid-containing rings in an aqueous solution containing 1% (*w*/*w*) BAC [31], and McConville et al. used an aqueous 1% (*w*/*v*) BAC solution for determining in vitro release of UC781, a topical microbicide from human and macaque-sized IVRs [38]. Besides applying artificial surfactants to increase API solubility in an aqueous dissolution medium, there are several other options for obtaining sink conditions. Various groups reported the addition of cyclodextrins for this purpose, and used a nonbuffered aqueous 1% 2-hydroxyl-propyl-*β*-cyclodextrin solution for drug release testing of IVRs containing anastrozole [71], levonorgestrel [83], and fixed-dose combinations of anastrozole and levonorgestrel [72]. The use of hydroalcoholic media is another means of increasing API solubility in the dissolution medium. Several research groups reported the use of hydroalcoholic/hydro-organic media for determining drug release from IVRs [34,37,38,40,43,44,47,59,63,64,65,75,76,77,78,79,80]. However, even though they might be the perfect choice for providing sink conditions for some IVRs that contain poorly soluble drugs, they also can cause significant problems and therefore their use should be well understood.

Some hydro-organic media are able to penetrate into polymers and cause polymer swelling. By doing so, they can alter the API diffusion characteristics in the polymer and thus impact overall drug release kinetics. In that case, they are unlikely to be applicable in QC, since drug release is no longer determined by the quality of the IVR, but dependent on the composition of the dissolution medium applied in the in vitro experiments. Therefore, when using hydro-organic release media, the degree of swelling should be determined and compared with that in pure aqueous media [34]. If the medium does not affect the release mechanism of the IVR, it can be applied for dissolution method design. Different organic solvents have been applied in hydro-organic release media. Helbling et al. used a water:ethanol mixture with an ethanol content of 20% (*v*/*v*) to determine progesterone release from EVA-IVRs [63,64,65]. McConville et al. determined drug release from UC781-loaded human-sized IVRs in a mixture of ethanol:water (1:1 *v*/*v*) [38]. For macaque-sized IVRs of the same type they used an isopropanol (IPA):water (1:1 *v*/*v*) mixture [38]. A particular reason for using two different types of solvents was not indicated by the authors, however due to the poor aqueous solubility of UC781, the use of IPA might have provided more reliable sink conditions. Overall, the use of IPA:water mixtures for screening drug release from IVRs was reported by many research groups [34,37,38,40,43,44,47,59,75,76,77,78,79,80]. Provided that the use of an organic solvent does not affect the general release mechanism of a drug-loaded IVR and the release rates obtained in water are proportional to those obtained in organic solvent:water mixtures, hydro-organic media also represents an appropriate means to accelerate drug release from polymer matrices. Externbrink et al. used mixtures of ethanol:water, IPA:water and acetonitrile:water with 25%, 50% or 75% organic solvent to accelerate drug release from a matrix-type IVR containing etonogestrel and ethinyl estradiol [34]. The release rate of both drugs increased with increasing solvent concentration, whereas with acetonitrile:water mixtures the strongest degree of acceleration was achieved. The same researchers also studied the impact of hydro-organic mixtures and temperature, and found out that combining a high organic solvent content and an increased media temperature resulted in the highest release rates for the formulations studied, without affecting the general release mechanism of the respective IVR [34].

As can be seen in the methods listed above, in many of the published in vitro QC test designs, no attention was given to properly address the composition and properties of vaginal fluid. However, some researchers tried, at least, to properly simulate vaginal pH-conditions. Woolfson et al. studied oxybutynin release from a silicone-based vaginal ring in a pH 4.0 (10 mM) acetate buffer [58]. Other research groups report the use of acetate buffers in a pH range of 4.0–4.5 [10,34,51,52,53,55,56,58]. A commonly used medium that well addresses average human vaginal fluid pH is 25 mM acetate buffer pH 4.2 [51,52,53,55]. Similarly, when the aim is to get an idea of vaginal drug release in rabbits, a species which is often used in the preclinical evaluation of vaginal ring segments, phosphate buffered saline (PBS, pH 7.40) represents a simple, but with regard to vaginal pH relevant medium, since rabbits typically have a neutral vaginal pH [73]. When addressing human vaginal pH conditions, acetate buffer is used as such or with some surfactant added when sink conditions cannot be obtained with the pure aqueous buffer medium. Surfactant concentrations vary between studies. Johnson et al. screened pyrimidinedione-loaded PU rings in 25 mM sodium acetate buffer pH 4.2, containing 0.05% or 2% Solutol^®^ HS 15 (poly-oxyethylene esters of 12-hydroxystearic acid, nowadays also known as Kolliphor^®^ HS 15) [74]. The buffer containing 0.05% Solutol^®^ HS 15 had been selected as the non-sink release medium in which the surfactant concentration was above the critical micelle concentration and therefore resulted in pyrimidinedione solubilization, i.e., a higher amount of pyrimidinedione dissolved than in the same volume of surfactant-free buffer. However, the effective surfactant concentration was too low to overcome pyrimidinedione saturation in the course of the experiments. By contrast, a concentration of 2% Solutol^®^ HS 15 resulted in sink conditions for the pyrimidinedione dose tested. The cumulative release profiles obtained in these two media were quite different. Under sink conditions, the IVR showed matrix-controlled release kinetics, whereas under non-sink conditions partition-controlled kinetics could be observed [74]. From these experiments, it could be clearly seen how the surfactant concentration can affect in vitro results obtained from IVRs containing poorly soluble compounds. The use of 2% Solutol^®^ HS 15 to provide sink conditions in experiments targeted on determining drug release of poorly soluble drugs from different types of IVRs was also reported by several other authors [53,73].

Using media with physiological vaginal pH represents a first setup towards physiologically relevant dissolution media for IVRs. Based on an intensive literature review in 1999, Owen and Katz introduced a “Vaginal Fluid Simulant” (VFS) that was developed to mimic properties and composition of human vaginal fluid [84]. VFS has a pH of 4.20 and is composed of: 3.51 g NaCl, 1.40 g KOH, 0.222 g Ca(OH)_2_, 0.018 g bovine serum albumin, 2.00 g lactic acid, 1.00 g acetic acid, 0.16 g glycerol, 0.4 g urea, and 5.0 g glucose. Although it is clear that this medium does not contain all ingredients of human vaginal fluid and also does not address the huge intra- and interindividual variability in vaginal fluid composition and properties, it represents the first step towards a more biorelevant test medium that can be applied in QC, and to some extent might be predictive for in vivo conditions. Therefore, it is currently widely used in IVR drug release testing. For this purpose, the medium is either used as such [21,22,35,40,68,69], or with slight modifications in osmolality [17,24,39,41,45,70]. Moreover, some authors added a preservative, such as sodium azide (0.1%) to VFS to prevent the growth of undesired bacteria and molds [49].

### 2.6. Entire Rings vs. Segments and Their Materials

During the early stage in development of an IVR, particularly for simply membrane- or matrix-based IVR types, it may be useful to screen a drug loaded polymer strand or just a part of an IVR. Determining drug release from IVR segments can minimize the amount of materials needed for fabrication (i.e., ring polymer, drugs and others), and reduces the amount of solvents/medium required for a drug release experiment. It is, thus, a valuable analytical approach to be applied in formulation development and screening, and is particularly attractive, when the final ring has not been manufactured yet.

When testing ring segments of reservoir, matrix or sandwich type IVRs, it is crucial to seal the ends of the segments to prevent drug release from the cut surface. Various techniques for sealing the segment ends have been described. These range from using the ring polymer [22,68,69] or a similar polymer [73] through dip coating of the cut surfaces in PVP or PVA solutions [57], to the use of an impermeable glue. To determine drug release from steroid containing IVR segments, a sealing made of Loctite^®^ and 1/16-inch thick polyethylene sheets proved to be impermeable for steroids [34,49,53,59,61,77]. In any case, sealing the ends of the segments must provide a release geometry equivalent to that of an entire ring. Thus, when testing drug release from ring segments, it is crucial to measure the exact length and diameter, as well as the mass of the segment, before starting the experiment. Clark et al. scaled their release rate values obtained from segments by extrapolating the surface area to that of a full-sized ring [53]. An alternative approach was used by Externbrink et al. who normalized release profiles obtained from segments to release of an entire IVR, based on the mass ratio (segment/ring) [34].

### 2.7. Temperature

With regard to the physiological conditions in the vaginal cavity, drug release tests should be performed at 37 °C. Whereas 37 °C is the “standard” test temperature applied in many dissolution test methods, for IVRs, various test methods applying a temperature of 25 °C have been described [17,24,39,41,70].

Externbrink et al. studied ENG release from the NuvaRing^®^ at different temperatures ranging from 37 °C to 55 °C [49]. The aim of their study was to investigate if temperature can be applied to accelerate drug release from membrane-type IVRs. For this purpose, a small volume USP apparatus 7 was used and IVR segments of ~1–1.5 cm length were tested. Length and mass of the entire IVR and each individual segment was measured, and drug release was normalized to the IVR mass. Real-time drug release was determined at 37 °C in 10 mL of VFS or water. Additional experiments were performed at 44, 50 and 55 °C to accelerate drug release [49]. The release profiles obtained at different temperatures were then examined for an Arrhenius relationship. Drug release rate increased with increasing temperature, but still followed zero-order kinetics. An Arrhenius relationship was achieved for rate constants calculated from real-time release and temperature-accelerated release profiles with adjusted sampling time points.

In another study, Externbrink et al. used the same test setup for assessing drug release from matrix-type IVRs at 37 °C and 45 °C [34]. Additionally, for this IVR type, higher drug release rates could be observed at elevated temperature.

Clark et al. investigated levonorgestrel diffusivity in different polyether urethane polymers (PEUs) at different temperatures, i.e., 23, 37 and 50 °C [73]. For this purpose, segments of 15 mm length were end-capped and the exact length, diameter and mass were determined. LNG diffusivity was calculated for each individual segment and increased with increasing temperature. Moreover, the extent of acceleration of drug release differed between the different PEUs.

In all of the cited experiments, it was shown that temperature is a critical parameter in dissolution method design, but also an appropriate means to accelerate drug release. Provided that a linear relationship can be established between real-time and accelerated drug release at increased temperatures, experiments performed at elevated temperatures can be an important tool for QC, since properly validated accelerated drug release experiments can be performed within a much shorter time than real time experiments.

### 2.8. Presentation of Data

In most of the publications cited in this review, results of drug release experiments are either presented as cumulative release, as daily release rate of the respective API, or both. To gain an idea, if the drug(s) is/are released from an IVR in a constant manner, presentation of the results as daily drug release can be of great advantage. By contrast, presentation of an average daily rate may underestimate essential details, for instance, a burst release immediately after application of the IVR or overestimate drug release in the phase of constant release. Sampling intervals can influence the proper detection of a burst release during the first hours or day(s) of drug release. Burst release phenomena have been observed for IVRs made of condensation-cure silicone and for reservoir-type IVRs during storage [58]. A burst release is characterized by an initial higher drug release rate compared to the claimed daily release rate.

As during an initial burst release, plasma concentrations can be much higher than the desired plasma concentration, and for certain drugs this can present with serious side effects or even toxic effects. Therefore, the burst release of an IVR should be well characterized. Thus, during the first 24–72 h of the experiment, an increased sampling frequency should be considered. After the initial phase of drug release, the sampling frequency can be reduced. However, as possible, daily release should be determined over the entire release period, since information on the release rates during different time periods (for example, day 1, 2–7 days, 8–14 days, 15–21 days, etc.) can be helpful for both developing discriminative QC methods or estimating in vivo drug release [44,57,67,71,83].

## 3. Discussion

By reviewing the official drug release methods available in international pharmacopoeias and guidelines, and the in vitro methods for assessing drug release of IVRs described in the literature, it became obvious that, to date, neither a standardized in vitro drug release method for QC, nor a biopredictive in vitro method for IVRs had been described. Various parameters that can affect drug release from an IVR are discussed in this review article. In vitro parameters include the apparatus design and the agitation rate, the volume, the composition and the temperature of the dissolution medium. Essential formulation parameters include the API solubility in the proposed test media as well as the IVR design. Finally, the analyst should be aware that the sampling schedule can have a significant impact on the obtained drug release profile.

Since the number of IVRs is likely to significantly increase within the near future, these systems should be monographed in international pharmacopoeias. Future research activities in assessing drug release of IVRs should focus on designing both biopredictive in vitro dissolution methods, as well as robust and discriminative QC methods that can be implemented in international pharmacopoeias and guidances.

Biorelevant test conditions should properly address the in vivo situation, i.e., the volume and composition of vaginal fluid [85] and the temperature in the vaginal vault. Attention should also be paid to the need of simulating changes in the vaginal microenvironment during the menstrual cycle, ageing or due to sexual intercourse. During sexual intercourse, human vaginal fluid mixes with human seminal fluid. This results in a temporary increase of vaginal pH due to the alkaline pH of human semen [86]. The pH increase will particularly affect drug release and dissolution of pH-sensitive drugs and a test setup simulating sexual intercourse should thus be considered when designing in vitro methods for IVRs that contain ionizable compounds. Human vaginal fluid contains various proteins [87]. Since biorelevant dissolution media are typically designed to reflect essential properties of the physiological fluids available at the site of drug administration/release, various simulated vaginal fluids contain proteins, for instance, albumin [7,84,88,89]. In drug release experiments with media containing proteins, drug-protein binding, which is, e.g., well known for steroids [30,90,91,92,93], should be properly addressed to capture the entire amount of drug released from the IVR. In such cases, the extent of protein binding should be evaluated in preliminary experiments by determining the recovery of different concentrations of an API dissolved in the simulated vaginal medium of interest. The use of biorelevant media can also present with challenges regarding microbial stability, since some biorelevant media provide excellent conditions for bacterial growth. Consequently, the addition of a preservative can be necessary to prevent biorelevant media from growth of undesired bacteria and molds. The selected preservative should be compatible with the other components of the medium and with the respective API. Overall, if biorelevant media are intended to be applied in in vitro drug release experiments of an IVR, the solubility and stability of the respective API should be investigated prior to the release experiments.

Currently, an increasing interest in developing IVRs can be observed. Formulation development of both generic IVRs and the design of novel IVR types would highly benefit from biopredictive in vitro dissolution methods. Since primary in vivo studies are often performed in animals such as rabbits [53,73,94], sheep [17,52,70], and macaques [10,17,24,37,38,39,40,45,71,74,79], appropriate in vitro models simulating the physiological environment in these animal species might also be required. McConville et al. tried to establish an IVIVC between in vivo data obtained in macaques and in vitro drug release data obtained with the macaque-sized IVR, but failed due to the lack of a predictive in vitro drug release method [38]. This is a clear indicator for the need of developing physiological relevant in vitro dissolution methods for these dosage forms. Recently, Boyd et al. presented an approach to better address intravaginal conditions in an in vitro dissolution experiment by applying a small volume of dissolution medium and mimicking vaginal tissue with a latex balloon [46]. This was another step forward in terms of a biorelevant test design, however, the proposed model is still far from a biopredictive in vitro model for IVRs.

Whereas most of the in vitro methods discussed in the present manuscript addressed a proper use of an IVR, human factors might be another fact to consider in future method designs. Independent of the IVR design and the drug(s) to be delivered, IVRs are designed to provide controlled drug release over extended periods of time and should ensure a safe and effective drug therapy and a good patient adherence. However, women’s use of a ring may deviate from perfect use [95]. The IVR might, for instance, be intentionally removed or be expelled periodically. Recently, Murphy et al. presented results of an in vitro study targeted on simulating the imperfect use of a dapavirine-releasing IVR [95]. They addressed the impact of systematic deviations (removal and storage of the IVR for different time periods) from a 28-day continuous-use protocol upon release performance. Furthermore, they assessed the effect of ring exposure to a range of common household chemicals and cosmetics, that in real-life conditions might be used for IVR cleansing or vaginal hygiene, on drug load and stability [95]. Even though the in vitro release experiments performed in the cited study were performed in a typical shake-flask setup and thus, cannot necessarily be regarded as biorelevant, the human factors addressed in this study might also be a facet to be considered in biorelevant test designs.

Particularly for drugs with limited solubility, the application of a biorelevant fluid volumes and composition in an in vitro experiment might not necessarily be the key for a successful prediction of the drug´s plasma level over the recommended time of use of the respective IVR. If typical vaginal fluid volumes will be used in a closed dissolution setup, such as e.g., the shake-flask setup, many drugs are likely to reach their saturation concentration within the first hours or days of the experiment. This will either stop further release or result in drug precipitation. Since in vivo drugs that had dissolved in vaginal fluid can be absorbed via the vaginal mucosa, drug concentration in the vaginal fluid will typically be much lower than in the in vitro setup, and the total amount of drug absorbed over the recommended time of IVR use is likely to be much higher than could be predicted from dissolution experiments run in such a closed dissolution system. Consequently, besides properly addressing vaginal fluid volume and composition, drug transport across the vaginal mucosa should also be considered in a biorelevant test design. Consequently, it might be worthwhile to evaluate dynamic or compartment models for this purpose. Based on these considerations, it will hopefully be possible to establish more biorelevant in vitro models in the near future.

In vitro dissolution methods applied in QC of IVRs should enable to distinguish between products/batches with the intended or undesired drug release. The use of a biorelevant test setup is, thus, not an essential requirement for method design. By contrast, QC methods are often characterized by a simple and robust test design with sufficient discriminatory power. Since IVRs represent dosage forms that release the API over a time range of weeks or even months, accelerated test methods can prove as a helpful tool in determining drug release and batch conformity of these systems. For developing accelerated in vitro drug release methods media, temperature or composition can be altered and elevated temperatures, the use of hydroalcoholic/hydro-organic solvents, or combinations thereof, have already been successfully applied to accelerate drug release from IVRs. A general prerequisite for applying such conditions is that they accelerate drug release, but do not alter the release (diffusion) mechanism [34]. To assess the impact of media composition on in vitro performance of the IVR, the swelling of an IVR should be recorded during the course of an in vitro release experiment to check whether the diffusion mechanism is affected by media composition or not. Moreover, for IVRs made of thermoplastic materials the temperature during in vitro release experiments should be strictly controlled [49], since even a small increase in temperature can increase the drug release rate. In QC, maintaining sink conditions is also of great importance to ensure that drug release is determined by the dosage form rather than by the in vitro test conditions. Sink conditions are a particular issue when assessing drug release from IVRs containing poorly soluble drugs, and can be achieved by either applying large volumes of dissolution media, or by adding surfactants to the dissolution media.

Unexpectedly, some experiments performed in the recent past have shown that the media volume can influence the drug release even when sink conditions are maintained [34]. Therefore, when dissolution experiments are performed in a closed system, such as the shake-flask setup, drug release should be determined in different media volumes to ensure the robustness and reliability of the test method. In experiments run with fixed media volumes, the renewal rate (sampling time points, sampling volumes) of the release medium can also have an impact on the drug release rate [46]. This impact should also be addressed in method design. A higher sampling frequency during the first day of the release experiment should be considered, when a burst release is expected to properly capture the burst release in the in vitro drug release profile.

Drug release testing of IVR segments can be helpful in formulation development or batch conformity testing when drug release of IVR segments is predictive for drug release of an entire ring. However, for method validation, studies with entire rings and ring segments should be performed to ensure the predictivity of release rates estimated from drug release experiments with ring segments, with regard to drug release of an entire IVR [34,49,53,73].

Due to expiring patent protections of existing IVRs, and the need for novel IVR formulations for HIV prevention and long-acting IVRs for contraception, hormone replacement therapy or treatment of endometriosis, regulatory agencies will be confronted with a variety of new drug applications. Currently, it does not seem to be clear what kind of in vitro data would be required for a successful application for an IVR, i.e., if cumulative or daily release should be plotted, if it is legal to calculate average daily release rates rather than providing detailed information on the variations in daily release over time, or if the chance for a burst release should be screened in more detail.

The information provided in the present review will, hopefully, be a platform for a more detailed discussion between academia, industry, and regulatory authorities on how robust and predictive in vitro drug release methods for IVRs should be designed to ensure safety and efficacy of these dosage forms.

## Figures and Tables

**Table 1:** Currently marketed intravaginal ring formulations (IVRs).

IVR	Indication	API	Status	Reference(s)
Estring^®^	hormone replacement	17β-Estradiol	marketed worldwide	[3]
Femring^®^	hormone replacement	17β-Estradiol-3-acetate	marketed worldwide	[3]
NuvaRing^®^ & generics	contraception	Ethinyl estradiol & etonogestrel	marketed worldwide	[3,4]
Progering^®^/Fertiring^®^	contraception	Progesterone	Peru and Chile	[3,4]
Annovera^®^	contraception	Ethinyl estradiol & segesterone acetate	FDA-approved	[5,6]

**Table 2:** Drug release method(s) for IVRs as provided in the FDA Dissolution Methods Database [32].

Drug	Apparatus	Speed	Medium	Volume	Sampling
Estradiol	Incubator shaker	130 rpm	0.9% saline	250 mL	1, 9, 16, 17, 18, 19, 45 d
Ethinyl estradiol & etonogestrel	Develop a method to characterize in vitro release				

**Table 3:** Overview of in vitro test methods for release testing of intravaginal rings (IVRs) described in the literature. VFS: vaginal fluid simulant (recipe by Owen and Katz), AB: sodium acetate buffer pH 4.2 or 4.5, IPA: isopropanol, EtOH: ethanol, PPB: potassium phosphate buffer pH 7.4, E: entire rings were tested, S: segments of IVRs were tested, R: rods were tested, MSR: macaque-sized ring, SSR: sheep-sized ring, FCP: flux controlled pump, PCL: polycaprolactone, PP: polypropylene, PEVA: polyethylene vinyl acetate, PLA: polylactide, PDMS: polydimethylsiloxane (silicone), PU: polyurethane, BSA: bovine serum albumin, SDS: sodium dodecylsulfate, and BAC: benzalkonium chloride.

Author(s)	API	Ring Material	Test Method	Formulation Tested	Test Medium	Agitation and Temperature
Asvadi et al. [21]	Acyclovir	PCL matrices as inserts for an IVR	Matrices were immersed in release medium in PP tubes	S	10 mL of VFS	Not specified
Externbrink et al. [34]	Ethinyl estradiol and etonogestrel	PEVA	Incubator shaker, USP apparatus 7 (400-DS)	E, S	AB and different hydro-organic media, 100 mL/10 mL (400-DS)	130 rpm/40 dpm, 37 and 45 °C
Wang et al. [35]	Isosorbide mononitrate, misoprostol	Silicone elastomer	Incubator shaker	E	50 mL VFS	60 rpm, 37 °C
Mc Conville et al. [36]	Tenofovir	PLA, PEVA	Incubator shaker	R	5 mL of VFS	60 rpm, 37 °C
Murphy et al. [37]	Dapivirine and darunavir	Silicone elastomer	Incubator shaker	MSR	100 mL of IPA:H_2_O (1:1) or VFS	60 rpm, 37 °C
McConville et al. [38]	UC781	Silicone elastomer	Incubator shaker	R, MSR	100 mL of EtOH:H_2_O (1:1) or 1% aqueous BAC solution; 50 or 100 mL of IPA:H_2_O (1:1)	60 rpm, 37 °C
Moss et al. [39]	Tenofovir disoproxil fumarate, emtricitabine, maraviroc	PDMS	Incubator shaker	MSR	100 mL VFS	60 rpm, 25 °C
Fetherston et al. [40]	MC1220	Silicone elastomer	Incubator shaker	MSR	50 mL VFS or 200 mL IPA:H_2_O (1:1)	60 rpm, 37 °C
Baum et al. [41]	Tenofovir and acyclovir	Silicone elastomer with PLA pods	Incubator shaker	E, S	100 mL VFS	60 rpm, 25 °C
Morrow et al. [42]	BSA and monoclonal antibody 2F5	Silicone elastomer	Incubator shaker	E	30 mL ammonium acetate buffer	60 rpm, 37 °C
Loxley et al. [43]	UC781	PEVA	Incubator shaker	E	100 mL IPA:H_2_O (1:1)	60 rpm, 37 °C
Boyd et al. [44]	Dapivirine and levonorgestrel	Silicone elastomer	Incubator shaker	E	50/200 mL IPA:H_2_O (1:1) (reservoir vs. matrix)	60 rpm, 37 °C
Srinivasan et al. [45]	Tenofovir disoproxil fumarate, emtricitabine and maraviroc	PDMS	Incubator shaker	MSR	100 mL VFS	60 rpm, 37 °C
Boyd et al. [46]	Disulfiram	PEVA	Incubator shaker	E	100 mL 2% SDS solution or 20 mL water	60 rpm, 37 °C
Boyd et al. [46]	Disulfiram	PEVA	Dual chambered release method, in an incubator shaker	E	Latex balloon with 20 mL of water, balloons were submerged in 100 mL of 2% SDS solution	60 rpm, 37 °C
Malcolm et al. [47]	TMC120	Silicone elastomer	Incubator shaker	E	200 mL IPA:H_2_O (1:1)	60 rpm, 37 °C
Malcolm et al. [31]	17β-Estradiol, 17β-Estradiol-3-acetate, metronidazole, norethisterone, norethisterone acetate, clindamycin, oxybutynin	Silicone elastomer	Incubator shaker	E	100 mL 1% aqueous BAC solution/ phosphate buffer	60 rpm, 37 °C
Mc Bride et al. [48]	5P12-RANTES	Silicone elastomer	Incubator shaker	E	15 mL Type 1 water	60 rpm, 37 °C
Externbrink et al. [49]	Ethinyl estradiol and etonogestrel	PEVA	USP apparatus 7 (400-DS)	S	10 mL VFS or water	37, 44, 50 and 55 °C, 40 dpm
Eder et al. [50]	Ethinyl estradiol and etonogestrel	PEVA	Incubator shaker	S	30 mL MilliQ water	130 rpm, 37 °C
Clark, J. et al. [51]	Tenofovir	PU	Incubator shaker	E	AB, volume not specified	80 rpm, 37 °C
Johnson et al. [52]	Tenofovir	PU	Incubator shaker	E	50 mL AB	80 rpm, 37 °C
Clark, M. et al. [53]	UC781	PU	Incubator shaker	S	100 mL AB or PPB containing 2% Solutol HS-15	80 rpm, 37 °C
Mesquita et al. [54]	Nonoxynol-9, acyclovir, tenofovir and tenofovir disoproxil fumarate	PU, PEVA, silicone elastomer	Incubator shaker	S	VFS, volume not specified	80 rpm, 37 °C
Teller et al. [55]	Dapivirine, maraviroc, tenofovir and tenofovir disoproxil fumarate, rhodamine B dextrane	PU with flux controlled pumps	Incubator shaker	FCPs	20 mL AB	80 rpm, 37 °C
Traore et al. [56]	Hydroxychloroquine	PU	Incubator shaker	MSR S	5 mL AB or 5 mL MRS broth	100 rpm, 37 °C
Chen et al. [57]	Hydroxychloroquine	PU	Incubator shaker	S	AB, volume not specified	100 rpm, 37 °C
Woolfson et al. [58]	Oxybutynin	Silicone elastomer	Incubator shaker	E	100 mL AB	100 rpm, 37 °C
Moss et al. [17]	Tenofovir and acyclovir	Silicone elastomer	Incubator shaker	SSR and S	100 mL VFS	110 rpm, 25 °C
Gupta et al. [59]	Dapivirine	PU ring with rods	Water bath shaker	S and E	5 mL 25:75 IPA:H_2_O, 50 mL 25:75 IPA:H_2_O	64 rpm, 37 °C
van Laarhoven et al. [60,61,62]	Ethinyl estradiol and etonogestrel	PEVA	Automated release control system	E	200 mL water	750 rpm, 37 °C
Helbling et al. [63,64,65]	Progesterone	PEVA	USP apparatus 1	E	1000 mL 20: 80 EtOH:H_2_O	25 rpm or 100 rpm, 37 °C
Verstraelen et al. [66]	Lactic acid	PEVA, Eudragit L 100	Incubation	S	5 mL demineralized water	37 °C, agitation not specified
Xia et al. [67]	Anastrozole	Silicone elastomer	USP apparatus 2	E	250 mL water	50 rpm, 37 °C
Pathak et al. [68]	Doxycycline	PCL matrices as inserts for an IVR	Incubation	S	10 mL VFS	37 °C, agitation not specified
Ugaonkar et al. [10]	MIV-150, levonorgestel, carrageenan, zinc acetate	PEVA	Incubator shaker	MSR	10 mL AB	37 °C, 100 rpm
Pathak et al. [22]	Metronidazole	PCL matrices as inserts for an IVR	Incubation	S	10 mL VFS	37 °C, agitation not specified
Dang et al. [69]	Tenofovir	PCL matrices as inserts for an IVR	Incubation	S	10 mL VFS	37 °C, agitation not specified
Moss et al. [70]	Tenofovir disoproxil fumarate and maraviroc	PDMS	Incubator shaker	SSR	100 mL VFS	60 rpm, 25 °C
Rotgeri et al. [71]	Anastrozole	PDMS	Incubator shaker	MSR	75 mL 1% aqueous hydroxy-propyl-β-cyclodextrin solution	100 rpm, 37 °C
Reinecke et al. [72]	Anastrozole and levonorgestrel	Not specified	Incubator shaker	Not specified	1% aqueous hydroxy-propyl-β-cyclodextrin solution, volume not specified	37 °C, agitation not specified
Clark, J. et al. [73]	Tenofovir and levonorgestrel	PU	Incubator shaker	E, S	2% Solutol in AB, volume not specified	80 rpm, 37 °C
Johnson et al. [74]	Pyrimidinedione	PU	Not specified	MSR	2% Solutol in AB or 0.05% Solutol in AB, volume not specified	Not specified
Murphy et al. [75]	Dapivirine and levonorgestrel	Silicone elastomer	Incubator shaker	E	200 mL IPA:H_2_O (1:1)	60 rpm, 37 °C
Johnson et al. [76]	Dapivirine and tenofovir	PU	Water bath shaker	S	5 mL 25:75 IPA:AB	64 rpm, 37 °C
Kaur et al. [77]	Dapivirine	PU	Water bath shaker	S	5 mL IPA:AB (25:75)	60 rpm, 37 °C
Fetherston et al. [78]	Dapivirine and maraviroc	Silicone elastomer	Incubator shaker	E	200 mL IPA:H_2_O (1:1)	60 rpm, 37 °C
